# BridgeSyn: a bridging fusion framework for drug combination synergy prediction

**DOI:** 10.1093/bib/bbaf624

**Published:** 2025-11-21

**Authors:** Qingyu Wang, Suwan Mao, Xiaoyan Liu, Quan Zou, Qing Liu, Xi Su, Junjie Wang, Wen Gao, Ximei Luo

**Affiliations:** Department of Medical Informatics, School of Biomedical Engineering and Informatics, Nanjing Medical University, 101 Longmian Avenue, Jiangning District, Nanjing, 211166 Jiangsu, China; Department of Medical Informatics, School of Biomedical Engineering and Informatics, Nanjing Medical University, 101 Longmian Avenue, Jiangning District, Nanjing, 211166 Jiangsu, China; Faculty of Computing, Harbin Institute of Technology, 92 West Dazhi Street, Nangang District, Harbin, 150001 Heilongjiang, China; Yangtze Delta Region Institute (Quzhou), University of Electronic Science and Technology of China, 1 Chengdian Road, Kecheng District, Quzhou, 324000 Zhejiang, China; Institute of Fundamental and Frontier Sciences, University of Electronic Science and Technology of China, 2006 Xiyuan Avenue, West Hi-tech Zone, Chengdu, 611731 Sichuan, China; Department of Anesthesiology, Hospital (T.C.M) Affiliated To Southwest Medical University, 182 Chunhui Road, Longmatan District, Luzhou, 646000 Sichuan, China; The Affiliated Foshan Women and Children Hospital, Guangdong Medical University, 11 Renmin West Road, Chancheng District, Foshan, 528000 Guangdong, China; Department of Medical Informatics, School of Biomedical Engineering and Informatics, Nanjing Medical University, 101 Longmian Avenue, Jiangning District, Nanjing, 211166 Jiangsu, China; Department of Oncology, The First Affiliated Hospital of Nanjing Medical University, 300 Guangzhou Road, Gulou District, Nanjing, 210029 Jiangsu, China; Institute of Fundamental and Frontier Sciences, University of Electronic Science and Technology of China, 2006 Xiyuan Avenue, West Hi-tech Zone, Chengdu, 611731 Sichuan, China

**Keywords:** drug synergy prediction, pretrained models, bridging fusion mechanism, combination therapy

## Abstract

Drug combination is a promising therapeutic strategy for complex diseases. However, only a small fraction of potential drug combinations exhibit true synergistic effects, making the prediction of drug synergy a critical yet challenging task. In this study, we propose BridgeSyn, a novel bridge fusion framework for drug synergy prediction. BridgeSyn leverages the knowledge from pretrained biological language models to enrich both drug compound and cell line representations. We introduce a bridging fusion mechanism that employs a set of shared latent tokens derived from global features, serving as a semantic interface to effectively fuse the representations of drug pairs and cell lines. By combining biological prior knowledge with this fusion strategy, BridgeSyn can capture complex biological interactions and achieve superior prediction results. Extensive experiments on two public datasets demonstrate that BridgeSyn consistently outperforms existing computation methods.

## Introduction

The strategic use of drug combinations has emerged as a cornerstone paradigm for treating complex diseases, including cancer [1], antimicrobial-resistant infections [2], and neurodegenerative disorders [3]. However, only a small fraction of drug combinations exhibit clinically significant synergistic effects. Therefore, there is a critical need to identify synergistic candidates within the vast combinatorial space. Traditional experimental screening methods, such as high-throughput combinatorial assays, are limited by prohibitive costs and low scalability. To address these limitations, computational approaches have demonstrated the potential to prioritize synergistic candidates. These methods typically follow a common pipeline: representing drugs and cell lines using numerical features, integrating these features through a fusion strategy, and then predicting synergy using a downstream model.

In computational drug combination synergy prediction, drug representations are primarily derived from molecular graphs, SMILES sequences, and physicochemical descriptors. Cell line characterization is predominantly anchored in multi-omics data, such as gene expression profiles, gene mutations, and copy number variations (CNVs). For instance, DeepSynergy [4] integrates chemical descriptors with gene expression profiles. Sidorov *et al.* [5] extended this framework by incorporating diverse chemical features, including structural fingerprints, MACCS keys, and ISIDA/SIRMS fragments. CCSynergy [6] leverages bioactivity profiles from the Chemical Checker, achieving improved accuracy and generalizability across a broad range of cell lines. PermuteDDS [7] further combines multiple drug fingerprints with two types of omics data, while SynergyX [8] demonstrates that the quantity of omics layers used (e.g. combining two or more) has a greater influence on prediction stability than the specific omics type. In parallel, recent advances in graph neural networks (GNNs) have fundamentally transformed drug representation learning and biological systems. For instance, transformer-based models have been developed to predict cancer genes by integrating multi-omics data with heterogeneous biological networks, achieving high interpretability and generalizability [9]. In the domain of drug discovery, knowledge graph-enhanced GNNs leverage large language models to generate rich molecular embeddings for drug–drug interaction prediction [10]. Beyond interaction prediction, novel variational Bayesian approaches have been proposed for scalable and interpretable clustering of attributed biological graphs [11]. For example, DeepDDS [12] and AttenSyn [13] utilize message-passing GNNs to encode molecular graphs into topology-aware embeddings. To enhance biological contextualization, some models incorporate auxiliary networks. GraphSynergy [14] embeds protein-protein interaction (PPI) topologies as prior knowledge . MFSynDCP utilizes a graph aggregation module with an adaptive attention mechanism to explicitly model the interaction mechanisms between drugs and cell lines, moving beyond simple feature extraction [15]. Furthermore, DD-PRiSM [16] explicitly models drug dosage as a continuous variable, recognizing its critical role in modulating synergistic effects. Despite these advances, most existing approaches have not sufficiently leveraged the rich contextual knowledge embedded in fully pretrained domain-specific models—such as UniMol [17] for molecular representations and ProtBERT [18] for protein/cell line features.

Beyond modeling drug and cell line features, a pivotal challenge lies in bridging their representations through advanced fusion strategies. Early methods such as DeepSynergy [4] and MatchMaker [19] predominantly relied on simplistic concatenation, whereas recent efforts explore more sophisticated fusion mechanism. For instance, SynergyX [8] and AttenSyn [13] employ multimodal mutual attention and attention-based pooling to integrate drug substructure features with omics data and cellular profiles. Similarly, DTSyn incorporates dual Transformer encoders to capture complex interactions, while DEML utilizes a hybrid ensemble layer combined with a task-specific fusion mechanism . DFFNDDS employs dual networks to fuse drug and cell line features . Notably, DGSSynADR pioneers multilayer perceptron (MLP)-based bilinear predictors that fuse dual-drug representations to enhance predictive performance. MDNNSyn employs multimodal fusion network layer with gated neural network for synergy score prediction. Despite these advances, the gap between the biological and data-driven characteristics of drugs and cell lines remains difficult to bridge, emphasizing the importance of tailored fusion strategies.

To overcome these challenges, we introduce BridgeSyn, a deep learning model that synergizes pretrained biological language models with a bridging fusion mechanism for drug combination synergy prediction. Specifically, BridgeSyn leverages UniMol to extract geometry-aware drug embeddings and ProtBERT to enrich gene expression profiles. Moreover, a fusion mechanism is designed to bridge the feature spaces of drug pairs and cell lines via a shared latent manifold, effectively serving as a semantic interface for drug combination data. Experimental evaluations in various settings demonstrate that BridgeSyn outperforms baseline models on two benchmark datasets. In addition, ablation studies confirm the effectiveness of each module and a case study further validates its performance in real-world scenarios.

## Materials and methods

We conducted a systematic assessment of BridgeSyn’s performance based on two widely benchmark datasets for drug combination synergy prediction: the O’Neil dataset [20] and the NCI-ALMANAC dataset [21]. The O’Neil dataset comprises 22 737 dose–response matrices quantified by Loewe additivity-based synergy metrics, encompassing 38 distinct therapeutic agents tested across 39 molecularly heterogeneous cancer cell lines. The NCI-ALMANAC database provides a larger-scale resource, containing 304 549 drug combination response profiles. It evaluates pairwise interactions between 104 FDA-approved drugs across the NCI-60 panel of extensively characterized tumor cell lines. Synergy assessments in NCI-ALMANAC are derived from the ComboScore metric, which jointly considers efficacy and potency.

### Problem formulation

**Figure 1:**
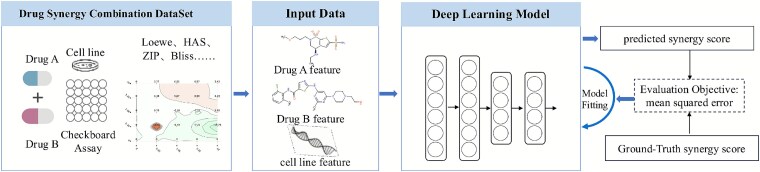
Schematic illustration of the problem setting.

In this work, the prediction of drug combination synergy is formulated as a regression problem, where a model learns to map representations of a drug pair and a cellular context to a continuous synergy score. Formally, the input is a triplet $(D_{A}, D_{B}, C)$, where $D_{A}$ and $D_{B}$ denote two distinct drugs, and $C$ represents a specific cancer cell line. The model outputs a scalar value $\hat{y} \in \mathbb{R}$, corresponding to the predicted synergy score of the drug combination. The objective is to learn a mapping function $f: (D_{A}, D_{B}, C) \rightarrow \hat{y}$ that accurately approximates the experimentally observed synergy. This function $f$ is generally implemented as a deep learning model. The model parameters are optimized by minimizing the discrepancy between the predicted synergy scores $\hat{y}$ and the ground-truth scores $y$ from the training dataset. Specifically, the learning process minimizes the mean squared error (MSE) loss, defined as: 


(1) \begin{align*}& L_{\text{MSE}} = \frac{1}{N} \sum_{i=1}^{N} (y_{i} - \hat{y}_{i})^{2}\end{align*}

where $N$ is the total number of training samples, $y_{i}$ is the true synergy score for the $i$th sample, and $\hat{y}_{i}$ is the score predicted by the model. For clarity, the mapping from the input triplet $(D_{A}, D_{B}, C)$ to the predicted synergy score $\hat{y}$ is schematically illustrated in Fig. 1.

### Overall structure

**Figure 2:**
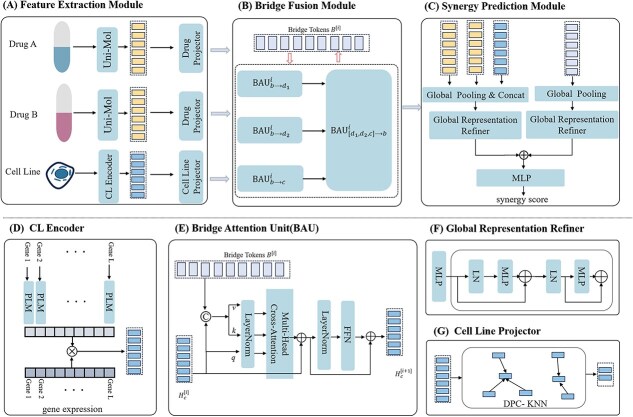
Schematic visualization of the BridgeSyn model: (A) Feature Extraction Module; (B) Bridge Fusion Module; (C) Synergy Prediction Module; (D) CL Encoder (in A); (E) Bridge Attention Unit(BAU, in B); (F) Global Representation Refiner (in C); (G) Cell Line Projector (in A).

This study proposes a novel fusion framework for predicting synergistic therapeutic interactions by effectively integrating drug pair and cell line information. As illustrated in Fig. 2, the architecture comprises three core components: a feature representation module, a bridge fusion module, and a synergy prediction module.

The drug pair is embedded using the pretrained Uni-Mol model, while the cell line representation is enriched through a ProtBert language model [18] applied to its gene expression profile. To facilitate joint modeling, both drug and cell line embeddings are projected into a shared feature space. Global pooling operations are then applied to these embeddings to generate bridge tokens that serve as semantic intermediaries between the two domains.

The bridge fusion module employs a Transformer decoder to model the complex interactions between drug pairs and cell lines via cross-attention, using the bridge tokens to guide information exchange. Finally, the fused representation is passed through an MLP to predict the synergy score of the drug combination.

### Drug and cell line feature

For drug representation, we first generate 3D conformers from their SMILES representation using RDKit. These conformers then serve as inputs to the Uni-Mol model to obtain drug embeddings $d_{1}, d_{2}$. The representation of cell lines is typically based on gene expression profiles. To enhance the representation of cell lines and capture more comprehensive biological information, we integrate protein sequence information with gene expression data. Specifically, each gene is represented $g_{i} = E_{i} \cdot P_{i}$, where $g_{i} \in \mathbb{R}^{ D_{P}}$ represents the final embedding of a given gene $i$, $E_{i}$ is the gene expression value, and $P_{i} \in \mathbb{R}^{ D_{P}}$ corresponds to the embedding for protein sequence based on the PLM. Finally, a cell line can be represented as $ G= \{g_{1}, g_{2},...,g_{L} \} \in \mathcal{R}^{L \times D_{P}}$ where $L$ is the number of genes.

### Drug projector

The drug pair embeddings from the pretrained Uni-Mol are individually projected into a shared feature space using a three-layer MLP, with shared weights across both drugs. Each MLP layer is followed by a layer normalization and a GELU activation. Formally, The projection function $f_{d} (\cdot )$ is defined as: 


(2) \begin{align*}\mathbf{h}^{(1)}&=\operatorname{GELU}\left(\operatorname{LN}\left(W_{1} \mathbf{d}+\mathbf{b}_{1}\right)\right)\nonumber \\[1.5mm] \mathbf{h}^{(2)}&=\operatorname{GELU}\left(\operatorname{LN}\left(W_{2} \mathbf{h}^{(1)}+\mathbf{b}_{2}\right)\right) \\[1.5mm] \hat{\mathbf{d}}&=\operatorname{GELU}\left(\operatorname{LN}\left(W_{3} \mathbf{h}^{(2)}+\mathbf{b}_{3}\right)\right)\nonumber\end{align*}

where $W_{i} \in \mathbf{R}^{d_{i} \times d_{i-1}}$ and $b_{i}$ are the learnable weights and biases of the $i$th MLP layer, LN denotes layer normalization, and GELU is the activation function. This projection is applied to each drug individually: 


(3) \begin{align*}& \hat{\mathbf{d}}_{1}=f_{\mathrm{drug}}\left(\mathbf{d}_{1}\right) \quad{\text{and}} \quad \hat{\mathbf{d}}_{2}=f_{\mathrm{drug}}\left(\mathbf{d}_{2}\right)\end{align*}

The resulting representations $\hat{\mathbf{d}}_{1}$ and $\hat{\mathbf{d}}_{2}$ are used in the subsequent fusion module.

### Cell line projector

The goal of the cell line projection module is to project the cell line representation $G \in \mathcal{R}^{L \times D_{P}}$ into a task-specific feature space with consistent dimensionality. To achieve this, we first apply a clustering-based cell line feature (CCF) encoder, which groups genes based on patterns in enriched gene expression, aiming to simulate biological pathways or functional modules. The resulting features are then transformed via a series of three MLPs to project them into a unified embedding space compatible with the drug representation.

The core concept of the CCF encoder is to employ a variant of the k-nearest neighbor-based density peaks clustering algorithm (DPC–KNN) [22] to partition the cell line feature $G$ into a predetermined number of clusters. For each cell line, the local density $\rho $ for the $i$th gene is compute based on its k-nearest neighbors in the feature space as follows: 


(4) \begin{align*}& \rho_{i}=\exp \left(-\frac{1}{k} \sum_{g_{j} \in \mathrm{KNN}\left({g}_{i}\right)} \left\|g_{i}-g_{j}\right\|_{2}^{2}, \right)\end{align*}

where $\mathrm{KNN}\left ({g}_{i}\right )$ denotes the k-nearest neighbors of gene $i$ in the feature space. $g_{i}$ and $g_{j}$ represent their enriched gene expression profiles.

Based on the computed local density for each gene, we calculate a distance indicator $\delta _{i}$, which is defined as the maximum distance between the feature vector of a gene and that of any other gene. Formally, the $\delta _{i}$ is defined as: 


(5) \begin{align*}& \delta_{i}=\left\{\begin{array}{@{}ll} \min_{j: \rho_{j}>\rho_{i}} \left\|g_{i}-g_{j}\right\|_{2}, & \text{ if } \exists\, j \text{ s.t. } \rho_{j}>\rho_{i} \\ \max_{j} \left\|g_{i}-g_{j}\right\|_{2}, & \text{ otherwise} \end{array}\right.\end{align*}

In order to determine the cluster centers, we first calculate a score for each gene by multiplying its $\rho _{i} $ and $ \delta _{i}$. The genes with the highest scores are then designated as the cluster centers. All other genes are subsequently grouped into the cluster whose centroid is closest in feature space, based on distance similarity. Finally, we utilize the average enriched gene expression profile within each cluster to represent the corresponding cell line as $G_{c} = \{ g_{1}^{c}, g_{2}^{c},...,g_{|C}^{c} \}$, where $|C|$ is the predefined cluster number, and $g_{i}^{c}$ is the cluster center.

By clustering enriched gene expression profiles, we effectively reduce the gene expression sequence length, which not only lowers computational cost but also highlights higher-order biological structure. The resulting grouped features are then passed through a stack of three MLPs. Each MLP layer consists of 1024 hidden units, followed by layer normalization and a GELU activation function to enhance nonlinearity and training stability. Finally, the cell line feature can be denoted as $G_{f} \in \mathcal{R}^{|C| \times 1024}$.

### Bridge fusion module

To facilitate effective interactions between drug pairs and cell line modalities, we propose a bridge fusion mechanism comprising two primary phases: bridge token generation and iterative bridge attention.

#### Bridge token generation

Initially, we derive comprehensive global representations from both drug pairs and cell lines using parallel global max-pooling, attention pooling and average-pooling operations. These aggregated features are then condensed into a fixed set of bridge tokens $B$, establishing a shared latent space for information exchange.

#### Iterative bridge attention

The core of our framework is the Bridge Attention Unit (BAU), which to fuse the drug and cell line features via the bridge tokens $B$. The BAU receives two sequences of features, denoted as $H_{m}$ and $H_{n}$. In specific, the calculation of $\operatorname{BAU}_{H_{m} \rightarrow H_{n}}^{[i]}\left (H_{m}, H_{n}\right )$ is as follows: 


(6) \begin{align*}& \begin{aligned} H_{n \rightarrow m}^{\prime} & =\operatorname{MHCA}\left(\operatorname{LN}\left(H_{m}\right), \operatorname{LN}\left( [H_{n},H_{m}]\right)\right)+H_{m} \\ H_{n \rightarrow m} & =\operatorname{FFN}\left(\operatorname{LN}\left(H_{n \rightarrow m}^{ \prime}\right)\right)+H_{n \rightarrow m}^{\prime }\end{aligned}\end{align*}

where LN denotes layer normalization, MHCA represents multihead cross attention, and FFN is the position-wise feed-forward network. In this operation, $H_{m}$ (e.g. a drug feature) is enhanced by integrating information from $H_{n}$ (e.g. the bridge tokens) via cross attention. An illustration of the BAU architecture is shown in Fig. 2E, where bridge tokens $B$, and cell line features $H_{c}$ are used as an example input pair.

For clarity, the transmission of features from layer $i$ to the subsequent layer $i + 1$ (across $L_{f}$ layers) is described as follows: 


(7) \begin{align*}& \begin{aligned} H_{d_{1}}^{[i+1]} & = \operatorname{BAU}_{b \rightarrow d_{1}}^{[i]}\left(H_{d_{1}}^{[i]}, B^{[i]}\right) \\ H_{d_{2}}^{[i+1]} & =\operatorname{BAU}_{b \rightarrow d_{2}}^{[i]}\left(H_{d_{2}}^{[i]}, B^{[i]}\right) \\ H_{c}^{[i+1]} & =\operatorname{BAU}_{b \rightarrow c}^{[i]}\left(H_{c}^{[i]}, B^{[i]}\right) \\ B^{[i+1]} & = \operatorname{BAU}_{ (d_{1}, d_{2}, c) \rightarrow g}^{[i]}\left(B^{[i]}, \left[H_{c}^{[i]}, H_{d_{2}}^{[i]}, H_{d_{1}}^{[i]}\right]\right) \end{aligned}\end{align*}

In this context, $H_{d_{1}}^{[i]}, H_{d_{2}}^{[i]}$, and $H_{c}^{[i]}$ denote the drug pair and cell line features at layer $i$, while $B^{[i]}$ represents the bridge tokens at the same layer. The terms $\operatorname{BAU}_{b \rightarrow d_{1}}^{[i]}\left (H_{d_{1}}^{[i]}, B^{[i]}\right )$, $\operatorname{BAU}_{b\rightarrow d_{2}}^{[i]}\left (H_{d_{2}}^{[i]}, B^{[i]}\right )$, and $\operatorname{BAU}_{b \rightarrow c}^{[i]}\left (H_{c}^{[i]}, B^{[i]}\right )$ signify the integration of the bridge tokens $B^{[i]}$ into $H_{d_{1}}^{[i]}, H_{d_{2}}^{[i]}$, and$H_{c}^{[i]}$ to generate the refined drug pair and cell line features. The terms, $\operatorname{BAU}_{ (d_{1}, d_{2}, c) \rightarrow b}^{[i]}\left (B^{[i]},[H_{c}^{[i]}, H_{d_{2}}^{[i]}, H_{d_{1}}^{[i]}]\right )$ indicates the bridge tokens, are updated by attending to the concatenated features from both drug pair and cell line.

The bridge tokens serve as an intermediary channel that not only carries global information but also mediates the exchange features, ensuring that relevant information flows seamlessly across modalities. By iteratively updating both the modality-specific features and the bridge tokens across $L$ layers, the bridge fusion mechanism ensures that information flows bidirectionally between the triplet. Acting as a mediator, the bridge tokens continuously refine and propagate information, leading to enriched and well-aligned representations that enhance the drug combination synergy prediction task.

Given the extensive feature-fusion operations integrated in BridgeSyn, we present a detailed analysis of its time complexity. Specifically, the total time complexity of the Bridge Fusion Module is given by: 


(8) \begin{align*}& O(L_{f} \cdot N_{b} \cdot D \cdot (N_{d} + N_{c}))\end{align*}

where $N_{d}$ is the number of atom-level tokens per drug, $N_{c}$ is the number of clustered cell features, $N_{b}$ is the number of bridge tokens, $D$ is the hidden dimension, and $L_{f}$ is the number of fusion layers. For a comprehensive breakdown of the derivation process, refer to Supplementary S2.

### Synergy prediction module

As the final stage of our framework, a dual-path prediction module is designed to effectively summarize and integrate the information from the input drug pair and cancer cell line. This module incorporates both bridge token-level interactions and modality-specific global summarization to enhance predictive performance.

Specifically, we first extract three global entity vectors corresponding to drug A, drug B, and the cell line, respectively, along with a fused representation derived from the bridge token embeddings. The three entity vectors are concatenated to form a global entity-level representation $ g_{\text{entity}} \in \mathbb{R}^{3d}$, which is then passed through an encoder $\mathcal{F}_{\text{entity}}(\cdot )$ based on the proposed global representation refiner (GRR). In parallel, the fused bridge representation is aggregated into $g_{\text{bridge}} \in \mathbb{R}^{d}$ and processed by a second encoder $\mathcal{F}_{\text{bridge}}(\cdot )$, also built upon the GRR design.

Each GRR encoder consists of multiple stacked residual blocks that follow a Transformer-inspired structure. Within each block, the first sublayer performs channel-wise mixing, preceded by layer normalization, and followed by a residual connection. The second sublayer includes a feedforward transformation with SwiGLU activation, a subsequent linear projection, and is further enhanced by LayerScale and DropPath regularization before another residual connection is applied.

The outputs of the two GRR branches are summed and fed into a linear prediction head $f_{\mathrm{pred}}(\cdot )$ to generate the final synergy score: 


(9) \begin{align*}& \hat{y} = f_{\mathrm{pred}}\left( \mathcal{F}_{\text{entity}}( g_{\text{entity}}) + \mathcal{F}_{\text{bridge}}(g_{\text{bridge}}) \right),\end{align*}

where $\hat{y}$ denotes the predicted synergy score. All learnable parameters in this module, along with the rest of the network, are optimized end-to-end using backpropagation with the objective of minimizing the MSE loss. A detailed summary of the algorithm is provided in Supplementary Algorithm S1.

## Results

### Experiment settings

We use four different data splitting settings follows the previous works to comprehensively evaluate the performance of our method and compared benchmarks, including DeepDDS [12], PermuteDDS [7], HypertranSynergy [23], HypergraphSynergy [24], DTF [25], CombFM [26], and Celebi’s method [27]. Initially, 10% of the samples are randomly reserved as a hold-out test set to evaluate the model’s generalization capability. The other 90% samples are split as follows:

- **Random-split**. The samples in the dataset are randomly partitioned into training, validation, and test sets.
- **Leave-cell-out**. The full set of cell lines was evenly divided into five subsets. In each iteration, samples associated with cell lines from four subsets were used for training, while the remaining subset was used for testing.
- **Leave-combination-out**. Drug combinations were randomly split into five equally sized folds. For each fold, data involving combinations from four-folds were used to train the model, and the remaining fold was reserved for testing.

We provide several performance measures, including MSE, $R$, and $R^{2}$. Detailed descriptions of these metrics are provided in Supplementary Section S3. Using multiple metrics, we obtain a more holistic understanding of the strengths and weaknesses of the model in predicting drug synergy. Descriptions of all baseline methods are included in Supplementary Section S4. For DeepSynergy, PermuteDDS, HypertranSynergy, and HypergraphSynergy, we used the official implementations released by the original authors with their recommended default hyperparameters. For DTF, ComboFM, and Celebi’s method, we adhered to the experimental protocols reported in the PermuteDDS study to ensure a consistent and fair comparison across all baselines.

### Hyper-parameter setting

The final architecture of BridgeSyn is defined by a set of tunable hyperparameters. Given the computational expense of performing exhaustive hyperparameter searches, we employed a grid-style search strategy to identify suitable configurations. Various architectural variants and parameter values were systematically evaluated. Hyperparameter tuning was conducted using five-fold cross-validation on O’Neil dataset. The optimal settings selected through this process are summarized in Table 1. Detailed information about the key submodules of the BridgeSyn is provided in Supplementary Table S7.

**Table 1:** Hyperparameter settings

Hyperparameter	Search space	Default value
Learning rate	$\{10^{-5},\,10^{-4},\,10^{-3},\,10^{-2}\}$	$10^{-4}$
Batch size	$\{32,\,64,\,128, 256\}$	256
Dropout rate	$\{0,\,0.1,\,0.2, 0.3\}$	0.2
Early stopping patience	$\{10,\,15,\,20,25\}$	25
Hidden units	$\{64,\,128,\,256,\,512,1024,2048\}$	1024
Optimizer type	$\{\mathrm{Adam},\, \mathrm{AdamW},\, \mathrm{Muon}\}$	Muon
The layer number of BAU	$\{1,\,2,\,3,\,4,5\}$	3

### Performance comparison

We conducted comprehensive experiments on the O’Neil and NCI-ALMANAC datasets to evaluate our proposed method, BridgeSyn, against seven benchmark approaches under different split settings. The results are summarized in Tables 2 and 3, where the best metric values are highlighted in bold.

**Table 2:** Benchmarking model performance on the O’Neil dataset

	Random split			Leave-cell-out			Leave-combination-out		
	RMSE	${R}^{2}$	PCC	RMSE	${R}^{2}$	PCC	RMSE	${R}^{2}$	PCC
BridgeSyn	**12.800**	**0.687**	**0.830**	19.912	0.226	0.529	**15.857**	**0.519**	**0.722**
HypertranSynergy	14.456	0.607	0.781	20.545	0.184	0.510	17.033	0.450	0.675
PermuteDDS	13.721	0.641	0.801	19.668	0.243	0.522	16.152	0.501	0.709
HypergraphSynergy	14.727	0.586	0.775	**19.537**	**0.252**	0.533	17.346	0.420	0.656
DeepSynergy	14.87	0.584	0.765	23.890	0.195	0.426	17.28	0.433	0.663
ComboFM	16.86	0.451	0.702	20.820	0.142	0.396	18.62	0.376	0.635
DTF	14.73	0.594	0.775	21.110	0.132	**0.535**	17.37	0.429	0.671
Celebi’s method	16.34	0.5	0.708	20.6	0.179	0.473	19.1	0.309	0.572

**Table 3:** Benchmarking model performance on the NCI-ALMANAC dataset

	Random split			Leave-cell-out			Leave-combination-out		
	RMSE	${R}^{2}$	PCC	RMSE	${R}^{2}$	PCC	RMSE	${R}^{2}$	PCC
BridgeSyn	**42.534**	**0.538**	**0.733**	54.251	0.247	**0.541**	**51.070**	**0.331**	**0.579**
HypertranSynergy	43.660	0.509	0.714	53.674	0.224	0.531	52.269	0.296	0.551
PermuteDDS	43.053	0.527	0.726	54.128	0.242	0.519	51.58	0.318	0.569
HypergraphSynergy	43.89	0.508	0.719	**53.398**	**0.273**	0.538	52.609	0.291	0.543
DeepSynergy	44.44	0.491	0.701	54.560	0.230	0.322	53.500	0.262	0.526
ComboFM	48.27	0.399	0.651	54.670	0.245	0.531	53.890	0.267	0.526
DTF	47.03	0.430	0.678	54.730	0.223	0.517	53.470	0.263	0.531
Celebi’s method	47.31	0.423	0.653	53.49	0.259	0.516	55.830	0.196	0.456

The results on the O’Neil benchmark (Table 2) demonstrate that BridgeSyn consistently outperforms all competing methods under both standard random splitting and more stringent extrapolation settings. In the random-split regime, BridgeSyn yields the lowest RMSE (12.800), highest $R^{2}$ (0.687), and highest Pearson correlation (0.830), indicating superior fit and predictive accuracy. Under the leave-cell–out split, HypergraphSynergy slightly edges out BridgeSyn in RMSE (19.537 versus 19.912) and $R^{2}$ (0.252 versus 0.226), but BridgeSyn remains highly competitive, suggesting robust generalization across unseen cellular contexts. Finally, in the leave-combination-out scenario, BridgeSyn again leads with RMSE 15.857, $R^{2}$ 0.519, and PCC 0.722, confirming its strength in extrapolating to novel drug combinations.

The evaluation on the larger NCI-ALMANAC dataset (Table 3) similarly highlights BridgeSyn’s scalability and predictive power. In the random split setting, BridgeSyn achieves the best RMSE (42.534), $R^{2}$ (0.538), and PCC (0.733), markedly improving over existing methods such as PermuteDDS and DeepSynergy. Under the leave-cell-out, HypergraphSynergy attains a marginally lower RMSE and higher $R^{2}$. In the leave-combination-out, BridgeSyn regains the lead (RMSE 51.070, $R^{2}$ 0.331, and PCC 0.579), underscoring its robust ability to predict synergy for unseen drug pairs in large-scale screening campaigns.

Across both benchmark datasets, BridgeSyn achieves state-of-the-art performance, especially under the random and cold-drug settings. The consistent improvements over baseline models such as DeepSynergy, PermuteDDS, and HypergraphSynergy highlight the effectiveness of incorporating advanced representation learning. In the leave-cell-out setting, HypergraphSynergy slightly outperforms other methods. This result may be explained by its auxiliary training objective, which focuses on reconstructing similarity networks for drugs and cell lines. Such a design likely enhances the model’s generalization ability when predicting on unseen cell lines. However, since this auxiliary task is trained using all available drugs and cell lines, the prediction module alone may still encounter entirely novel drug–cell line pairs during inference. Despite this, BridgeSyn achieves comparable results, with only a minor performance gap. It is important to note that even the best-performing method under the leave-cell-out regime yields an $R^{2}$ value <0.5. This suggests that current models are not yet reliable enough for real-world deployment. The reduced performance in this setting underscores a key challenge in the field. Improving generalization to unseen cellular contexts remains an open problem, and future research may benefit from incorporating more comprehensive omics profiles or prior biological knowledge such as signaling pathways.

We further evaluated its performance on independent external test sets, with the corresponding results summarized in Table 4, where the best metric values are highlighted in bold. On the O’Neil dataset, BridgeSyn achieves the best performance in terms of $R^{2}$ (0.677) and PCC (0.829), outperforming all compared methods, while obtaining a competitive RMSE (14.744), i.e. close to the best (13.183 by HypertranSynergy). On the NCI-ALMANAC dataset, BridgeSyn achieves the lowest RMSE (42.816), indicating a superior error-reducing capability, while its $R^{2}$ (0.496) and PCC (0.705) are competitive with the best-performing model HypertranSynergy ($R^{2}$ = 0.516 and PCC = 0.719). Overall, the results demonstrate that BridgeSyn consistently delivers strong and balanced performance across multiple metrics and datasets, highlighting the effectiveness of the proposed bridging fusion mechanism, and the utilization of pretrained biological language models.

**Table 4:** Benchmarking model Performance on the independent dataset

	O’Neil			NCI-ALMANAC		
	RMSE	$R^2$	PCC	RMSE	$R^2$	PCC
BridgeSyn	14.744	**0.677**	**0.829**	**42.816**	0.496	0.705
PermuteDDS	15.144	0.659	0.821	43.338	0.484	0.696
HypergraphSynergy	16.710	0.585	0.788	43.730	0.474	0.693
HypertranSynergy	**13.183**	0.641	0.805	43.588	**0.516**	**0.719**
DeepSynergy	16.840	0.578	0.765	45.325	0.435	0.670
ComboFM	16.080	0.541	0.754	46.370	0.457	0.685
DTF	16.150	0.548	0.752	49.860	0.372	0.700
Celebi’s method	16.500	0.529	0.728	45.860	0.469	0.688

To rigorously assess the statistical significance of our model’s performance improvements, we conducted paired t-tests on repeated cross-validation folds for each scenario and dataset. The resulting $P$-values, which quantify the probability that the observed performance difference occurred by chance, are presented in Supplementary Tables S1, S2, and S3. Our model’s performance gains were most pronounced in the random split scenario, where it achieved statistically significant improvements ($P <.05$, and often $P <.01$) over the majority of competitors. In the more challenging leave-combination-out setting, BridgeSyn maintained significant advantages over DeepSynergy and DTF. However, in the leave-cell-out scenario, the improvements over methods like HypertranSynergy and PermuteDDS did not reach statistical significance, reflecting the inherent difficulty of generalizing to entirely new cell lines.

### Ablation studies

Our model, BridgeSyn, incorporates two primary contributions: (i) the integration of prior knowledge through pretrained drug and protein models, and (ii) a novel bridge fusion architecture designed to facilitate effective interaction between drug and cell line representations. To quantitatively assess the contribution of each component, we conducted an ablation study by systematically modifying or removing key modules within BridgeSyn. The following model variants were evaluated:

- BridgeSyn(a): Replaces the proposed bridge fusion mechanism with a full transformer model that directly processes concatenated drug and cell line features, without the bridging structure.
- BridgeSyn(b): Eliminates pretrained protein language model knowledge, utilizing only gene expression profiles for cell lines.
- BridgeSyn(c): Employs solely one-hot encoded SMILES representations for drugs.
- BridgeSyn(d): Combines one-hot encoded SMILES representations of drugs with gene expression data of cell lines.
- BridgeSyn(e): Represents the most ablated version, removing both core contributions by using only one-hot encoded SMILES for drugs and gene expression for cell lines, with fusion performed by a plain transformer instead of the bridge fusion module.

**Figure 3:**
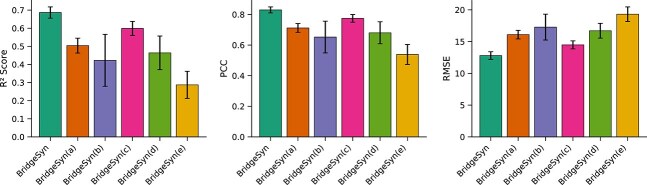
Bar charts on ablation experiments on the O’Neil dataset under random split setting.

**Figure 4:**
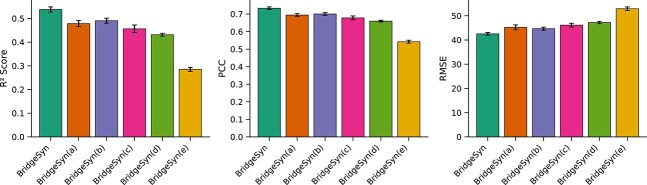
Bar charts on ablation experiments on the NCI-ALMANAC dataset under random split setting.

As shown in Figs 3 and 4, each core component of BridgeSyn contributes meaningfully to the overall model performance. The removal of the bridge fusion module led to a marked performance drop, highlighting that our proposed fusion paradigm captures cross-modal interactions between drugs and cell lines more effectively than naïve concatenation-based approaches. Further degradation was observed in BridgeSyn(b) and BridgeSyn(c), where pretrained protein and drug representations were individually excluded. Interestingly, BridgeSyn(d), which combines one-hot SMILES and gene expression features, outperformed BridgeSyn(b) and BridgeSyn(c) individually. However, its performance still lagged behind the full model, suggesting that a single-modality representation cannot fully capture the complex biological underpinnings of drug response. The poorest performance was observed in BridgeSyn(e), where both key components were removed. This result confirms that these two innovations are not only individually beneficial but also complementary, jointly enabling BridgeSyn to accurate synergy prediction.

To rigorously evaluate the benefit of the proposed bridge fusion module, we implemented several representative fusion baselines under the same encoder backbone and predictor. The detailed descriptions of representative fusion baselines can be found in Supplementary S6. Supplementary Table S4 reports the performance of different fusion mechanisms. The results clearly and consistently demonstrate the superiority of our proposed Bridge Fusion. Our Bridge Fusion mechanism consistently achieved the best performance across both datasets and all metrics. Its success can be attributed to several key design principles. Firstly, it facilitates an efficient and focused interaction. Unlike Cross-Attention, which processes all tokens collectively, the bridge tokens act as a set of task-oriented queries. They proactively extract salient features from both drugs and the cell line, avoiding information overload and guiding the model to focus on the interactions most critical for synergy prediction. Secondly, the bridge provides a symmetric and flexible space for all modalities to interact, avoiding the potentially unbalanced, pairwise exchanges seen in co-attention mechanisms, while allowing for adjustable model capacity.

We have conducted additional ablation experiments comparing DPC–KNN with k-means, DBSCAN. As shown in Supplementary Table S5, although all clustering methods provide performance improvements compared to using raw features, DPC–KNN consistently yields the best results on both O’Neil and NCI-ALMANAC datasets, demonstrating its superior ability to capture biologically meaningful structure in gene expression data. We also performed a sensitivity analysis on the number of clusters, which is a key hyperparameter of DPC–KNN. As shown in Supplementary Table S5, the model exhibits stable performance across a broad range of cluster numbers (8–128), with only slight variations metrics. This indicates that our method is robust to reasonable changes in clustering granularity, reducing the burden of hyperparameter tuning in practical applications.

### Drug contribution analysis

To quantify the contribution of each constituent drug in a combination therapy, we conducted both perturbation-based and gradient-based attribution analyses. For perturbation analysis, we measured the change in predicted synergy after replacing one drug with a null baseline embedding (zero or mean drug embedding). This yielded a direct estimate of each drug’s relative importance. For gradient-based attribution, we employed integrated gradients (IG) to obtain token-level attributions, which we aggregated to compute per-drug contributions. The precise mathematical formulations of these methods, including $\Delta S_{a}$, $\Delta S_{b}$, and IG attributions, are provided in the Supplementary Materials.

**Figure 5:**
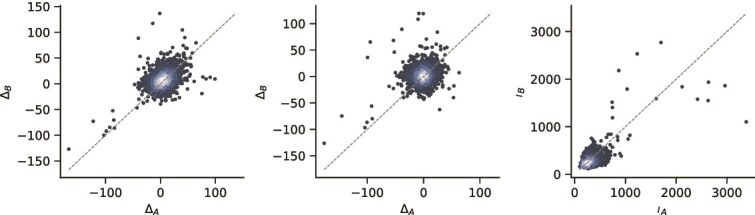
The scatter plot of drug contribution.

As shown in Fig. 5, the vast majority of points cluster tightly around the diagonal line, indicating that our model predominantly predicts synergy through balanced, cooperative contributions from both drugs—consistent with the biological principle of true pharmacological synergy. Nevertheless, a small but consistent subset of combinations deviates from the diagonal, revealing cases where one drug’s contribution is dominant.

### Order sensitivity analysis of drug pairs

We evaluated the sensitivity of our model’s predictions to the sequential order of input drug pairs. For every drug–drug–cell line triplet, we created two distinct inputs: one with the order (Drug A and Drug B) and another with (Drug B and Drug A), both paired with the identical cell line. This experiment aimed to determine if our model produces consistent synergy predictions when the sequence of the drug pair is inverted.

**Figure 6:**
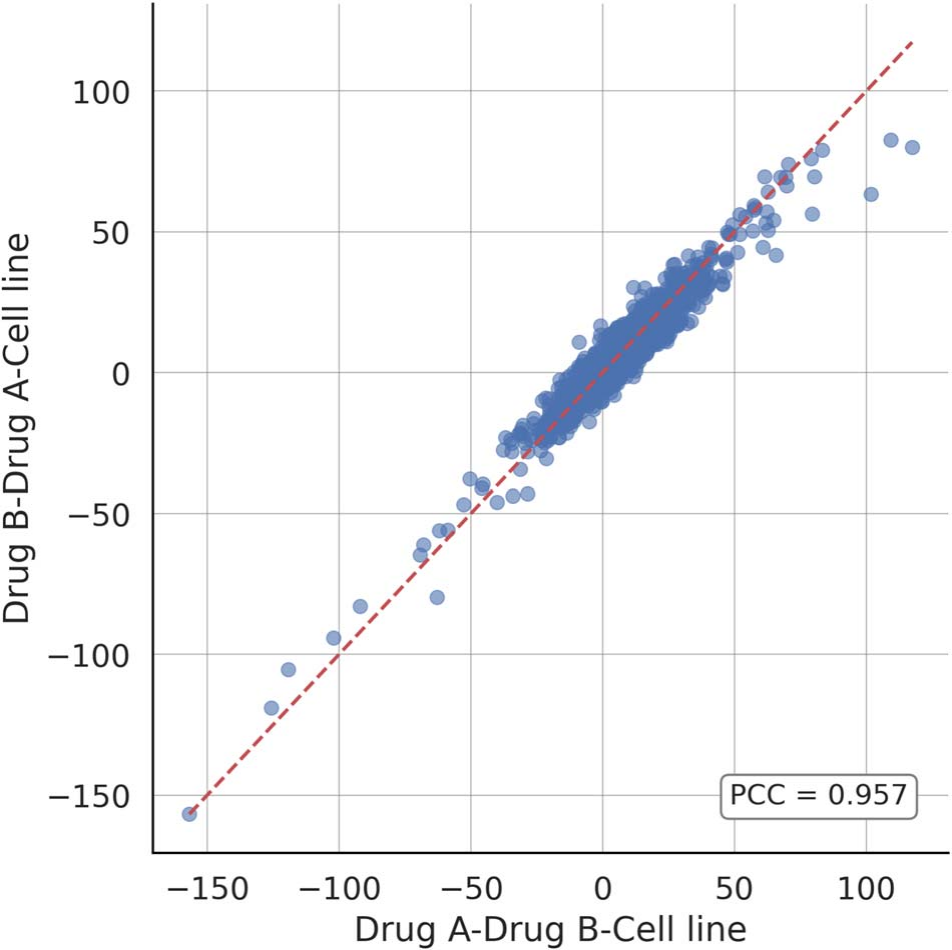
Scatter plot of synergy scores predicted from two different input sequence of drug pairs.

Figure 6 presents a scatter plot of the predicted synergy scores for both input orientations using the O’Neil independent test set. Each point represents a unique drug–cell line pair, with the x-axis corresponding to predictions from the (Drug A and Drug B) order and the y-axis from the (Drug B and Drug A) order. The dashed line indicates perfect symmetry and serves as a reference for ideal consistency . As shown in the figure, the majority of points are symmetrically distributed around the identity line, indicating strong agreement between the two input orders. Quantitatively, the Pearson correlation coefficient between the two prediction sets is 0.957, reflecting a high degree of linear consistency. These results demonstrate that our model is largely invariant to drug pair order, producing stable and reliable predictions regardless of sequence.

### Predictions aggregated by tissue

**Figure 7:**
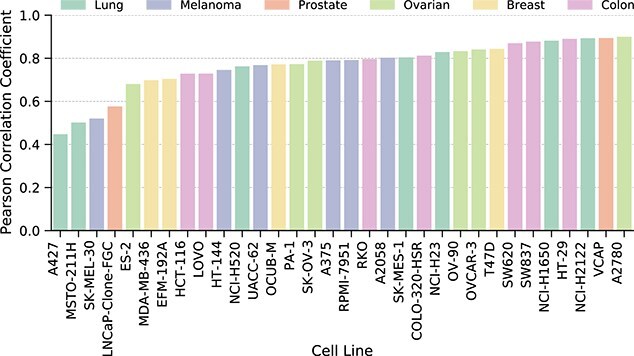
Pearson correlation coefficients between model predictions and experimental scores across 31 cancer cell lines.

To evaluate how well the model generalizes across different biological contexts, we computed the PCC between predicted and experimental drug response scores for each of the 31 cancer cell lines in the independent test set. Figure 7 visually summarizes these results, with each bar representing a cell line’s PCC, color-coded by tissue type. The observed PCC values, spanning from 0.47 to 0.91, highlight the model’s generally strong predictive capability across a majority of cell lines, albeit with noticeable variations tied to tissue origin.

A visual inspection of the figure reveals substantial variation in prediction accuracy across tissues. For example, cell lines from Colon and Breast tissues showed consistently high correlations, with most achieving PCCs >0.75. In contrast, predictions for some Melanoma and Lung cell lines were markedly more variable. While certain cell lines in these groups (e.g. HT-144, UACC-62, and NCI-H1650) had PCCs >0.80, others such as SK-MEL-30 and A427 fell well <0.60.

To further quantify this phenomenon, we analyzed the intra-tissue consistency of model predictions by computing the standard deviation of PCC values within each tissue. This revealed that Lung and Melanoma tissues exhibited the highest variability, with standard deviations of 0.150 and 0.138, respectively. In contrast, Colon and Breast tissues were the most consistent, with standard deviations of 0.059 and 0.056, indicating more uniform model performance across their respective cell lines. The Ovarian and Prostate groups showed intermediate levels of variability (SD = 0.094 and 0.129).

This intra-tissue variability may pose a challenge for clinical translation, where heterogeneity within patient subtypes is common. Future work may benefit from incorporating uncertainty estimation, hierarchical tissue models, or domain adaptation techniques to better capture the range of biological responses within each tissue type.

### Case study

To further validate the applicability of BridgeSyn in real-world scenarios, we trained BridgeSyn on all validated drug–drug–cell line triplets from the NCI-ALMANAC dataset and used it to predict synergy scores for the remaining triplets lacking experimentally measured synergy data. Table 5 highlights several high-scoring predictions that are supported by prior studies in the literature. These results demonstrate several of the top predictions, such as the combinations of Paclitaxel with Gefitinib and Docetaxel with Tamoxifen citrate, have already been reported to enhance therapeutic efficacy in preclinical or clinical studies, further validating the predictive power of our model.

**Table 5:** The novel synergistic drug combinations supported by the literature

**Drug 1**	**Drug 2**	**Cell line**	**Tissue**	**Predicted score**	**Reference**
Paclitaxel	Gefitinib	OVCAR-3	Ovarian	214.989	[28]
Tamoxifen citrate	Docetaxel	SK-MEL-2	Skin	185.31	[29]
Paclitaxel	Vinorelbine tartrate	DU-145	Prostate	177.868	[30]
Docetaxel	Gefitinib	HL-60	Promyeoloblast	159.021	[31]
Docetaxel	Gefitinib	NCI-H23	Lung	108.8324	[32]
Altretamine	Paclitaxel	OVCAR-3	Ovarian	150.854	[33]
Paclitaxel	Gefitinib	A549	Lung	93.08	[34]
Gefitinib	Dasatinib	IGROV-1	Ovarian	81.187	[35]

## Conclusion

This work presents BridgeSyn, which leverages a pretrained compound encoder to capture the structural and geometric characteristics of drug pairs. To represent cell lines, we design a novel encoding strategy that fuses transcriptomic profiles with contextualized features extracted from a PLM, yielding biologically meaningful and information-rich cell representations. These heterogeneous inputs are unified through a custom-designed fusion module, where global representations from drug–cell–drug triples act as intermediary bridge tokens. A cross-attention mechanism is then applied to enable dynamic interaction modeling by allowing these bridge tokens to exchange information with the individual components of the triplet. Comprehensive experiments show that BridgeSyn consistently outperforms baseline methods across multiple benchmark datasets, underscoring its robustness and practical utility in accelerating drug synergy discovery.

Despite the strong results, several future directions remain. One is the integration of multi-omics modalities of cell lines [36, 37]. Another is extending the model from predicting a scalar synergy score to generating a detailed interaction landscape, offering finer-grained insights into combination effects across dose ranges.

**Key Points**

- A deep learning model named BridgeSyn is proposed for the prediction of drug combination synergy, employing newly designed fusion mechanisms and leveraging pretrained models trained on biological sequences.
- We introduce a pretrained protein language model augmented with cell line-specific gene expression data, leading to substantial improvements in prediction accuracy.
- BridgeSyn demonstrates competitive and robust performance across multiple data split settings on two public datasets, validating its effectiveness and generalizability.

## Supplementary Material

supplementary_material_for_bridge_syn_bbaf624

Table_S1_bbaf624

Table_S2_bbaf624

Table_S3_bbaf624

Table_S4_bbaf624

Table_S5_bbaf624

Table_S6_bbaf624

Table_S7_bbaf624

## Data Availability

The data and code from this study are openly accessible on GitHub (https://github.com/IILab-Resource/BridgeSyn.git).
