# Supplementary material for: BridgeSyn: a bridging fusion framework for drug combination synergy prediction
Source: Brief Bioinform. 2025 Nov 21;26(6):bbaf624. doi: 10.1093/bib/bbaf624 (PMC12636530; doi:10.1093/bib/bbaf624)
Supplement: supplementary_material_for_bridge_syn_bbaf624 [file supplementary_material_for_bridge_syn_bbaf624.pdf]

BridgeSyn: A Bridging Fusion Framework for Drug Combination Synergy Prediction: supplemental document

This document provides supplemental material for the main article.

S1. THE PSEUDOCODE OF BRIGESYN

Algorithm S1. BridgeSyn: Drug Combination Synergy Prediction

Require:
SMILES$_A$: SMILES string of Drug A
SMILES$_B$: SMILES string of Drug B
GeneExpr $\in \mathbb{R}^L$: Gene expression profile of the cell line (length L)
ProteinSeqs $= \{P_1, P_2, ..., P_N\}$: Protein sequences corresponding to the N genes
Ensure:
$\hat{y}$: Predicted synergy score

▷ **1. Feature Representation**
$d_1 \leftarrow$ UniMol(Generate3DConformer(SMILES$_A$)) ▷ Drug A embedding
$d_2 \leftarrow$ UniMol(Generate3DConformer(SMILES$_B$)) ▷ Drug B embedding
for $i = 1$ to N **do**
    $P_i \leftarrow$ ProtBERT(ProteinSeqs[i])[[CLS]] ▷ Get [CLS] token embedding
    $g_i \leftarrow$ GeneExpr[i] $\times P_i$ ▷ Enriched gene embedding
$G \leftarrow \{g_1, g_2, ..., g_N\} \in \mathbb{R}^{N \times D_P}$ ▷ Cell line representation
▷ **2. Feature Projection**
$\hat{d}_1 \leftarrow f_{\text{drug}}(d_1)$ ▷ Shared MLP projector for drugs
$\hat{d}_2 \leftarrow f_{\text{drug}}(d_2)$
$G_c \leftarrow$ CCF Encoder(G) ▷ Cluster genes using DPC-KNN
$G_f \leftarrow f_{\text{cell}}(G_c)$ ▷ MLP projector for cell line
▷ **3. Bridge Token Generation**
$B^{(0)} \leftarrow$ GlobalPool$([\hat{d}_1, \hat{d}_2, G_f])$ ▷ Concatenate and apply Max/Avg/Attn Pooling, then project to $N_b \times D$
▷ **4. Bridge Fusion (Iterative for L layers)**
$H_{d1}^{(0)} \leftarrow \hat{d}_1$, $H_{d2}^{(0)} \leftarrow \hat{d}_2$, $H_c^{(0)} \leftarrow G_f$
for $i = 0$ to $L - 1$ **do**
    $H_{d1}^{(i+1)} \leftarrow \text{BAU}(B^{(i)}, H_{d1}^{(i)})$ ▷ $B \rightarrow d1$
    $H_{d2}^{(i+1)} \leftarrow \text{BAU}(B^{(i)}, H_{d2}^{(i)})$ ▷ $B \rightarrow d2$
    $H_c^{(i+1)} \leftarrow \text{BAU}(B^{(i)}, H_c^{(i)})$ ▷ $B \rightarrow c$
    $B^{(i+1)} \leftarrow \text{BAU}([H_{d1}^{(i+1)}, H_{d2}^{(i+1)}, H_c^{(i+1)}], B^{(i)})$ ▷ $(d1, d2, c) \rightarrow B$
▷ **5. Synergy Prediction**
$g_{\text{entity}} \leftarrow \text{Concat}(\text{GlobalPool}(H_{d1}^{(L)}), \text{GlobalPool}(H_{d2}^{(L)}), \text{GlobalPool}(H_c^{(L)}))$
$g_{\text{bridge}} \leftarrow \text{GlobalPool}(B^{(L)})$
$\hat{y} \leftarrow f_{\text{pred}}\left(F_{\text{entity}}(g_{\text{entity}}) + F_{\text{bridge}}(g_{\text{bridge}})\right)$ ▷ GRR encoders and final MLP
return $\hat{y}$

S2. THE COMPLEXITY OF THE BRIDGE FUSION MODULE

Given the extensive feature-fusion operations in BridgeSyn, we provide a detailed time complexity analysis. The computational bottleneck lies primarily in the Bridge Fusion Module, which

employs iterative cross-attention operations via the Bridge Attention Unit.

The BAU unit performs multi-head cross-attention (MHCA) between two sequences. The time complexity of MHCA is dominated by the computation of the attention matrix and is generally $O(N_q \cdot N_k \cdot D)$, where N_q and N_k denote the lengths of the Query and Key/Value sequences, respectively, and D is the hidden dimension.

Within one BAU layer, four distinct cross-attention operations are performed:

1. $B \rightarrow d1$: Bridge tokens attend to the atom-level representations of drug 1.

$$\text{Complexity} = O(N_b \cdot N_d \cdot D)$$

2. $B \rightarrow d2$: Bridge tokens attend to the atom-level representations of drug 2.

$$\text{Complexity} = O(N_b \cdot N_d \cdot D)$$

3. $B \rightarrow c$: Bridge tokens attend to cell line clusters.

$$\text{Complexity} = O(N_b \cdot N_c \cdot D)$$

4. $(d1, d2, c) \rightarrow B$: The concatenated features of both drugs and the cell line attend to the bridge tokens.

$$\text{Complexity} = O(N_b \cdot (2N_d + N_c) \cdot D)$$

Summing the four operations for a single layer yields:

$$O(2 \cdot (N_b \cdot N_d \cdot D) + (N_b \cdot N_c \cdot D) + (N_b \cdot (2N_d + N_c) \cdot D)) = O(N_b \cdot D \cdot (4N_d + 2N_c)) \,.$$

Since this process is repeated for L fusion layers, the total complexity of the Bridge Fusion Module is:

$$O(L \cdot N_b \cdot D \cdot (N_d + N_c))$$

where N_d is the number of atom-level tokens per drug, N_c is the number of clustered cell features, N_b is the number of bridge tokens, D is the hidden dimension, and L_f is the number of fusion layers.

S3. EVALUATION METRICS

To evaluate the performance of proposed model, we employ several widely used metrics for regression tasks, including the Root Mean Squared Error(RMSE), the Coefficient of Determination(R^2) and Pearson's Correlation Coefcient(PCC). These metrics are formulated as follows:

$$\text{RMSE} = \sqrt{\frac{1}{n}\sum_{i=1}^{n}(y_i - \hat{y}_i)^2} \tag{S1}$$

$$R^2 = 1 - \frac{\sum_{i=1}^{n}(y_i - \hat{y}_i)^2}{\sum_{i=1}^{n}(y_i - \bar{y})^2} \tag{S2}$$

$$\text{PCC} = \frac{\sum_{i=1}^{n}(y_i - \bar{y})(\hat{y}_i - \bar{\hat{y}})}{\sqrt{\sum_{i=1}^{n}(y_i - \bar{y})^2}\sqrt{\sum_{i=1}^{n}(\hat{y}_i - \bar{\hat{y}})^2}} \tag{S3}$$

where n denotes the number of samples, y_i represents the ground truth value of the i-th sample and $\hat{y}_i$ is the corresponding predicted value. The $\bar{y}$ and $\bar{\hat{y}}$ indicate the mean of ground truth values and the mean of predicted values, respectively.

S4. BASELINES

For each baseline, we now summarize its core architecture, input features, and fusion mechanism. This provides readers with sufficient context to understand the methodological differences and the significance of our improvements.

- DeepSynergy: Integrates chemical descriptors of drugs with gene expression profiles of cell lines. The two modalities are concatenated and processed through a deep neural network for synergy prediction.
- PermuteDDS: Represents drugs with multiple fingerprint types and cell lines with two omics modalities. Its key innovation is a permutable feature fusion network that effectively integrates heterogeneous data sources.
- HypertranSynergy: Employs a hypergraph transformer to model drug–drug–cell interactions, leveraging a granularity-level fusion strategy to predict anticancer drug synergy.
- HypergraphSynergy: Builds upon multi-way relation-enhanced hypergraph representation learning. It further incorporates an auxiliary similarity-network reconstruction task to improve generalization across drugs and cell lines.
- DTF (Deep Tensor Factorization): Uses a tensor factorization framework combined with deep learning to capture latent interactions and predict synergistic effects.
- ComboFM: Exploits factorization machines to systematically model multi-way interactions among drugs and cell lines, enabling preclinical drug combination prediction.
- Celebi's method: Utilizes multi-omics data from cell lines in an in-silico framework designed for the prediction of synergistic anticancer drug combinations.

S5. STATISTICAL SIGNIFICANCE OF BRIDGESYN VS. OTHER METHODS

Table S1. Statistical significance (p-values) of BridgeSyn vs. other methods on the O'Neil dataset.

	Random split			Leave-cell-out			Leave-combination-out		
	RMSE	R^2	PCC	RMSE	R^2	PCC	RMSE	R^2	PCC
HypertranSynergy	1.2e-02	7.7e-03	1.1e-02	9.6e-01	7.2e-01	9.9e-01	2.2e-01	2.4e-02	3.4e-02
PermuteDDS	4.8e-02	3.8e-02	4.4e-02	9.3e-01	9.2e-01	6.2e-01	6.8e-01	3.8e-01	3.4e-01
HypergraphSynergy	8.6e-03	7.8e-03	1.8e-02	9.6e-01	7.2e-01	9.9e-01	2.2e-01	2.4e-02	3.4e-02
DeepSynergy	1.9e-04	3.2e-04	6.2e-04	3.8e-01	5.3e-02	1.3e-01	1.9e-02	1.3e-04	4.7e-04
DTF	1.0e-04	4.5e-05	4.9e-05	9.4e-01	9.8e-01	5.9e-01	5.6e-02	5.9e-04	6.2e-04

Table S2. Statistical significance (p-values) of BridgeSyn vs. other methods on the NCI-ALMANAC dataset.

	Random split			Leave-cell-out			Leave-combination-out		
	RMSE	R^2	PCC	RMSE	R^2	PCC	RMSE	R^2	PCC
HypertranSynergy	2.6e-03	5.3e-04	5.4e-03	7.9e-01	3.1e-01	8.6e-01	6.6e-01	1.9e-01	2.0e-01
PermuteDDS	1.2e-01	6.0e-02	7.3e-02	9.9e-01	8.8e-01	2.5e-01	8.1e-01	3.3e-01	3.3e-01
HypergraphSynergy	2.6e-03	5.3e-04	5.4e-03	7.9e-01	3.1e-01	8.6e-01	6.6e-01	1.9e-01	2.0e-01
DeepSynergy	2.3e-04	8.8e-05	4.4e-04	3.0e-01	7.3e-03	2.4e-02	9.3e-02	1.9e-02	2.1e-03
DTF	3.5e-05	8.5e-06	1.9e-05	3.8e-01	1.3e-02	5.4e-03	2.5e-01	1.4e-03	1.6e-03

Table S3. Statistical significance (p-values) of BridgeSyn vs. other methods on the independent dataset.

	O'Neil			NCI-ALMANAC		
	RMSE	R2	PCC	RMSE	R2	PCC
PermuteDDS	5.7e-01	5.5e-01	4.9e-01	8.7e-01	8.8e-01	1.0e+00
HypergraphSynergy	1.3e-02	2.5e-02	2.5e-02	5.0e-02	3.1e-02	2.7e-02
HypertranSynergy	**6.7e-03**	1.0e-01	4.5e-02	1.2e-04	**1.5e-02**	**9.6e-01**
DeepSynergy	3.3e-03	2.5e-03	1.5e-03	4.2e-05	4.1e-05	3.5e-04
DTF	8.1e-04	4.6e-04	4.8e-04	4.7e-07	5.4e-07	3.7e-06

S6. ABLATION STUDY ON FUSION MECHANISMS

To rigorously evaluate the benefit of the proposed bridge fusion module, we implemented several representative fusion baselines under the same encoder backbone and predictor.

Late Fusion. Given pooled embeddings of the two drugs and the cell line, the features are concatenated and passed through an MLP:

$$z = \mathrm{MLP}\big(\mathrm{Concat}\big(\mathrm{Pool}(H_{d1}), \mathrm{Pool}(H_{d2}), \mathrm{Pool}(H_c)\big)\big), \tag{S4}$$

where $\mathrm{Pool}(\cdot)$ denotes mean pooling. This mechanism aggregates features only at the representation level, without explicit token-level interaction.

Cross-Attention Fusion. A set of learnable bridge tokens $B^{(0)} \in \mathbb{R}^{N_b \times D}$ iteratively attend to all modalities through multi-head cross-attention:

$$B^{(l+1)} = \mathrm{MHCA}\Big(Q = B^{(l)}, K = [H_{d1}, H_{d2}, H_c], V = [H_{d1}, H_{d2}, H_c]\Big), \quad l = 0, \dots, L_f - 1. \tag{S5}$$

The final $B^{(L_f)}$ is pooled and fed into the prediction head.

Gated Fusion. Let $\bar{H}_{d1}, \bar{H}_{d2}, \bar{H}_c$ be pooled features. A gating network produces adaptive weights:

$$[g_1, g_2, g_3] = \sigma(W \cdot [\bar{H}_{d1}; \bar{H}_{d2}; \bar{H}_c]), \tag{S6}$$

$$z = g_1 \cdot \bar{H}_{d1} + g_2 \cdot \bar{H}_{d2} + g_3 \cdot \bar{H}_c, \tag{S7}$$

where $\sigma(\cdot)$ is the element-wise sigmoid.

Co-Attention Fusion. Drugs and cell line embeddings are refined by mutual attention:

$$\tilde{H}_d = \mathrm{MHCA}(Q = H_d, K = H_c, V = H_c), \quad \tilde{H}_c = \mathrm{MHCA}(Q = H_c, K = H_d, V = H_d). \tag{S8}$$

Final representations are obtained by pooling $\tilde{H}_d$ and $\tilde{H}_c$ and concatenating with the second drug's pooled representation.

Table S4 reports the performance of different fusion mechanisms. The results clearly and consistently demonstrate the superiority of our proposed Bridge Fusion. Our Bridge Fusion mechanism consistently achieved the best performance across both datasets and all metrics. Its success can be attributed to several key design principles. Firstly, it facilitates an efficient and focused interaction. Unlike Cross-Attention, which processes all tokens collectively, the bridge tokens act as a set of task-oriented queries. They proactively extract salient features from both drugs and the cell line, avoiding information overload and guiding the model to focus on the interactions most critical for synergy prediction. Secondly, the bridge provides a symmetric and flexible space for all modalities to interact, avoiding the potentially unbalanced, pairwise exchanges seen in co-attention mechanisms, while allowing for adjustable model capacity.

Table S4. Comparison of different fusion mechanisms on the independent dataset. Best results are highlighted in **bold**.

	O'Neil			NCI-ALMANAC		
	RMSE	R2	PCC	RMSE	R2	PCC
Bridge Fusion	**14.744**	**0.677**	**0.829**	**42.816**	**0.496**	**0.705**
Cross-Attention Fusion	15.636	0.636	0.801	48.095	0.364	0.623
Gated Fusion	17.133	0.563	0.758	43.533	0.479	0.692
Co-Attention Fusion	18.927	0.467	0.689	43.536	0.479	0.692
Late Fusion	17.235	0.558	0.755	52.605	0.239	0.512

S7. CLUSTERING METHOD JUSTIFICATION AND SENSITIVITY ANALYSIS

We adopted DPC-KNN because it determines cluster centers based on local density and relative distance, which is particularly well aligned with the biological characteristics of gene expression data. We have conducted additional ablation experiments comparing DPC-KNN with k-means, DBSCAN. As shown in Table S5, although all clustering methods provide performance improvements compared to using raw features, DPC-KNN consistently yields the best results on both O'Neil and NCI-ALMANAC datasets, demonstrating its superior ability to capture biologically meaningful structure in gene expression data.

We also performed a sensitivity analysis on the number of clusters, which is a key hyperparameter of DPC-KNN. As shown in Table S6, the model exhibits stable performance across a broad range of cluster numbers (8 to 128), with only slight variations metrics. This indicates that our method is robust to reasonable changes in clustering granularity, reducing the burden of hyperparameter tuning in practical applications.

Table S5. Performance comparison of different clustering methods in CCF encoder on benchmarking datasets.

	O'Neil			NCI-ALMANAC		
	RMSE	R2	PCC	RMSE	R2	PCC
DBSCAN	13.473	0.653	0.809	42.858	0.531	0.729
K-means	13.586	0.648	0.806	42.622	0.536	0.733
DPC-KNN	**12.800**	**0.687**	**0.830**	**42.534**	**0.538**	**0.733**

S8. DRUG CONTRIBUTION ANALYSIS

To estimate the contribution of each constituent drug in a combination (D_a, D_b, C), we performed a perturbation-based analysis. Let S_{full} denote the predicted synergy score using both drugs. We then replace the embedding of one drug with a null baseline (either a zero vector or the mean embedding of all drugs) while keeping the other unchanged:

$$\Delta S_a = S_{\text{full}} - S_{\text{w/o}\,a}, \quad \Delta S_b = S_{\text{full}} - S_{\text{w/o}\,b}. \tag{S9}$$

The normalized attribution for drug D_a is defined as

$$\Delta_A = \frac{|\Delta S_a|}{|\Delta S_a| + |\Delta S_b| + \epsilon}, \tag{S10}$$

where ϵ is a small constant to ensure numerical stability.

Table S6. Sensitivity analysis of the number of clusters in DPC-KNN on benchmarking datasets.

	O'Neil			NCI-ALMANAC		
	RMSE	R2	PCC	RMSE	R2	PCC
cluster no=8	13.162	0.669	0.818	42.371	0.542	0.736
cluster no=16	13.243	0.665	0.816	42.452	0.531	0.729
cluster no=32	12.800	0.687	0.830	42.534	0.538	0.733
cluster no=64	13.167	0.669	0.819	42.313	0.541	0.735
cluster no=128	12.991	0.677	0.824	42.291	0.542	0.737

To complement perturbation analysis with a gradient-based approach, we employed Integrated Gradients (IG). For a model F and input embeddings (D_a, D_b), IG attributes the prediction difference between a baseline (D'_a, D'_b) and the actual input as

$$\mathrm{IG}_i(x) = (x_i - x'_i) \times \int_{\alpha=0}^{1} \frac{\partial F(x' + \alpha(x - x'))}{\partial x_i}\, d\alpha. \tag{S11}$$

In practice, the integral is approximated with n steps along the linear interpolation path. For each drug, we aggregate the absolute attributions over all embedding dimensions:

$$I_a = \sum_j |\mathrm{IG}_{a,j}|, \quad I_b = \sum_j |\mathrm{IG}_{b,j}|. \tag{S12}$$

S9. MODEL ARCHITECTURE

Table S7. Detailed architecture key submodules. LN = LayerNorm, GELU = Gaussian Error Linear Unit, DP = Dropout.

Component	Operation / Layers	Key Parameters
**Cell Line Encoder**	DPC-KNN + MLPs	Input dim = 1024, Output dim = 1024, sample ratio = 32
**Drug Encoder**	MLPs	Input dim = 512, Output dim = 1024
**MLPs**	3-layer MLP with LN + GELU + DP after each hidden layer	Hidden dim = 1024, DP = 0.1
**Pooling**	Global attention pooling + Reduce (max/mean)	Applied to drug1, drug2, cell line embeddings
**Bridge Tokens**	Concatenation of pooled features	9 pooled vectors (max, mean, attn × 3 modalities)
**Bridge Fusion Layers**	Stacked BAU	Depth = 3, each with 3 branches (Drug1, Drug2, Cell)
**BAU**	Cross-attention between context and bridge tokens	embed_dim = 1024, n_{heads} = 32, head_dim = 32
**Cross-attention**	LN → Linear Q/K/V projections → scaled dot-product attention → output proj	$Q, K, V \in \mathbb{R}^{bsz \times seq \times d}$, softmax stabilization
**Global Pooling**	Reduce (max + mean)	Applied to updated drug, cell, and fusion tokens
**Global Representation Refiner**	Global Representation Refiner	Input dim = 1024
**Global Representation Refiner**	Global Representation Refiner	Input dim = 3072
**Prediction Head**	Fully connected layer	Linear(1024 → 1)
